# Substrate binding induces structural changes in cytochrome P450cam

**DOI:** 10.1107/S1744309108044114

**Published:** 2009-01-31

**Authors:** Keisuke Sakurai, Hideo Shimada, Takashi Hayashi, Tomitake Tsukihara

**Affiliations:** a Institute for Protein Research, Osaka University, Suita 565-0871, Japan; b Picobiology Institute, Graduate School of Life Science, University of Hyogo, Koto 3-1-1, Kamigori-cho, Ako-gun, Hyogo 678-1205, Japan; c Department of Applied Chemistry, Graduate School of Engineering, Osaka University, Suita 565-0871, Japan

**Keywords:** cytochrome P450cam, (+)-camphor, redox potential

## Abstract

The binding of (+)-camphor to cytochrome P450cam (P450cam) expels a cluster of waters at the active site, raising the redox potential of the haem to an extent that allows reduction by the electron-transfer system. This binding was reported to involve no significant structural changes in the protein. Here, two ferric P450cam structures partially complexed with (+)-camphor were determined by X-­ray crystallography at 1.30–1.35 Å resolution, revealing the structures of the substrate-free and substrate-bound forms. (+)-Camphor binding induces rotation of Thr101 to form a hydrogen bond that acts as a hydrogen donor to a peripheral haem propionate. This bonding contributes to the redox-potential change.

## Abstract 2

X-ray structures of ferric cytochrome P450cam partially complexed with the substrate (+)-camphor to two different extents were determined at 1.30–1.35 Å resolution, revealing the protein structures of the substrate-free and substrate-bound forms.

## 1. Introduction

Cytochrome P450cam (P450cam) is a thiolate haem-containing monooxygenase that catalyzes the regioselective and stereoselective hydroxylation of (+)-camphor to produce 5-*exo*-hydroxycamphor (Gunsalus *et al.*, 1974 ▶). The reaction is initiated by the binding of (+)-camphor to the resting state of the enzyme. Substrate binding changes the spin state of the haem iron from low to high and raises its redox potential (Fe^3+^/Fe^2+^ couple) by ∼100 mV (Gunsalus *et al.*, 1974 ▶; Sligar & Gunsalus, 1976 ▶), which allows the reduction of the enzyme by an electron-transfer system comprising NADH-putidaredoxin reductases and putidaredoxin. X-ray crystallographic studies on substrate-free (Poulos *et al.*, 1986 ▶) and substrate-bound (Poulos *et al.*, 1987 ▶) forms of the enzyme at 2.2 and 1.6 Å resolution, respectively, revealed that a cluster of waters (six molecules) at the active site of the substrate-free form, one of which binds to the haem iron, are expelled from the active site upon binding of (+)-camphor. This occurs without an accompanying conformational change of the protein apart from a slight shift (by about 0.3 Å) of a phenylalanine residue near the bound substrate. These crystallographic results explain the change in spin state and entropy-driven substrate binding (Griffin & Peterson, 1972 ▶). Recent X-ray structures of the substrate-bound form at 1.4–1.6 Å resolution (Schlichting *et al.*, 2000 ▶; Meilleur *et al.*, 2005 ▶) demonstrate that Thr101 forms a hydrogen bond to the haem 6-­propionate, which differs from the previously observed conformation (Poulos *et al.*, 1987 ▶). The formation of a hydrogen bond between Thr101 and the haem 6-­propionate is important because it raises the redox potential of the haem iron. However, whether the conformational change of Thr101 is tightly coupled to the substrate binding remains unknown. To understand the mechanisms of the large redox-potential changes, elimination of the water cluster from the active site and other events that take place upon substrate binding, a higher resolution X-ray structure is necessary for the substrate-free form.

Here, we provide X-ray structures of the substrate-free and substrate-bound forms of the enzyme at 1.30–1.35 Å resolution. Substrate binding induces hydrogen-bond formation between Thr101 and the ionized haem 6-propionate side chain. This hydrogen bond may significantly increase the redox potential of the haem.

## 2. Materials and methods

### 2.1. Preparation of protein

Wild-type P450cam was expressed in *Escherichia coli* strain JM109 and was purified to homogeneity by SDS–PAGE according to pre­viously described procedures (Harada *et al.*, 2008 ▶). The enzyme samples were dissolved in 50 m*M* potassium phosphate buffer pH 7.4 containing 50 m*M* KCl and 1 m*M* (+)-camphor, frozen in liquid nitrogen and stored at 193 K until use. Enzyme samples with an absorption ratio of *A*_391_/*A*_280_ > 1.6 were used. Concentrations of the wild-type protein were spectrophotometrically determined using an extinction coefficient of 102 m*M* ^−1^ cm^−1^ at 391 nm (Gunsalus & Wagner, 1978 ▶).

### 2.2. Crystallization

Crystals of the ferric form of P450cam were grown using the sitting-drop vapour-diffusion method. 3–5 µl P450cam solution (30 mg ml^−1^ P450cam, 250 m*M* KCl, 10 m*M* dithioerythritol, 1 m*M* (+)-camphor) was mixed with an equal volume of the reservoir solution [50 m*M* Tris–HCl pH 7.4, 250 m*M* KCl, 10 m*M* dithioerythritol, 1 m*M* (+)-camphor and 22–30%(*w*/*v*) PEG 4000] and allowed to stand at 268 K for 2 d.

### 2.3. Camphor soaking

Crystals of ferric P450cam grown in the crystallization buffer were transferred to a reservoir solution saturated with (+)-camphor and allowed to stand at 268 K for 1 d. The (+)-camphor-saturated solution was prepared by adding excess fine powdered (+)-camphor to the reservoir solution and stirring extensively for 1 d. Undissolved (+)-­camphor was removed from the solution.

### 2.4. X-ray experiments and structure determination

After the crystals had been equilibrated with a solution containing 20%(*v*/*v*) 2-methyl-2,4-pentandiol (MPD), they were frozen in liquid nitrogen. X-ray diffraction data for crystals that had or had not been soaked in (+)-camphor-saturated buffer were collected on beamlines BL41XU and BL44XU at SPring-8, respectively. The diffraction images were indexed, integrated, scaled and merged using the programs *HKL*-2000 and *SCALEPACK* (Otwinoski & Minor, 1997 ▶).

Initial phasing of the crystals was performed by molecular replace­ment using the structure of ferric P450cam (PDB code 2cpp) as a reference molecule and the program *MOLREP* (Collaborative Computational Project, Number 4, 1994 ▶). The crystal structures were refined using the program *REFMAC* (Collaborative Computational Project, Number 4, 1994 ▶). Difference Fourier maps were calculated with (*F*_o_ − *F*_c_)exp(2π*i*α_c_) coefficients to detect the (+)-camphor molecule at the active site, where *F*_o_ and *F*_c_ were the observed and calculated structure amplitudes, respectively, and α_c_ was the calculated phase. The refined structures were inspected using the program *PROCHECK* (Laskowski *et al.*, 1993 ▶).

## 3. Results and discussion

**Figure 1:**
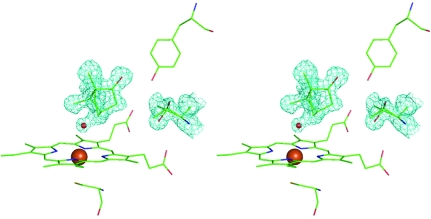
Stereoview of the structure of the active site and its vicinity in ferric cytochrome P450cam determined from the unsoaked crystals. Active-site residues (Cys357 and Tyr96), haem (iron represented by a large orange sphere), substrate (+)-camphor, a water molecule (a small red sphere) liganded to haem and Thr101 are shown as stick models (with green C atoms, red O atoms and a blue N atom). (+)-Camphor, the water molecule and Thr101 are represented by electron density from the composite OMIT map (cyan, contoured at 1.5σ). The Thr101 side chain exhibits two rotamer structures. (Note the two red sticks extended in different directions: one toward the hydroxy group of Tyr96 and the other toward the peripheral haem 6-propionate. The electron density of the latter is much higher than that of the former.) It is also noted that the electron density of the keto group of (+)-camphor is much stronger than that of the bound water. Figures were drawn using *PyMOL* (v.0.99rc6).

**Figure 2:**
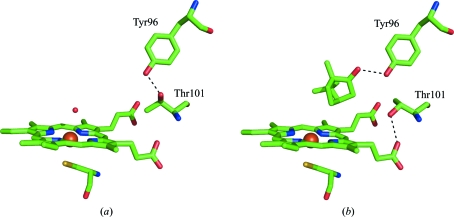
Structures of the active-site and the vicinity of the minor (*a*) and major (*b*) component of ferric cytochrome P450cam represented by stick models with green C atoms and a red O atom. (*a*) A water molecule bound to haem iron is shown by a red sphere. Thr101 is hydrogen bonded to Tyr96. (*b*) Tyr96 and Thr101 are hydrogen bonded to (+)-camphor and peripheral haem 6-propionate, respectively. Dotted lines indicate hydrogen bonds. Figures were drawn using *PyMOL* (v.0.99rc6).

Crystals of ferric P450cam were grown in crystallization buffer con­taining 1 m*M* (+)-camphor and the crystals grown were soaked in crystallization buffer saturated with (+)-camphor (∼8 m*M*). The structures of the soaked and unsoaked ferric P450cam crystals were solved at 1.30 Å (PDB code 2zwt) and 1.35 Å (PDB code 2zwu) resolution, respectively (Table 1 ▶). The structure of the unsoaked P450cam shows an active site that is partially occupied by (+)-­camphor and a water molecule liganded to the haem iron and rotamers of Thr101 (Fig. 1 ▶). The water molecule bound to the haem iron is 1.56 Å from the C5 atom of (+)-camphor. Hence, the water does not coexist with (+)-camphor in one protein structure, indicating that the crystals are a mixture of (+)-camphor-bound and (+)-camphor-free forms. It was noted that the electron density arising from the keto group of (+)-­camphor was much higher than that of the bound water, suggesting that the (+)-camphor-bound form is the major component and the water-bound form is the minor component. The two rotamers of the Thr101 side chain also showed unequal electron density; the form with the hydroxy group directed toward the peripheral haem 6-­propionate showed much higher electron density than the form with the hydroxy group directed toward Tyr96. The structures of the minor and major components are depicted in Figs. 2 ▶(*a*) and 2 ▶(*b*), respectively. In the soaked P450cam, the population of the major component increased, while the minor component decreased.

**Figure 3:**
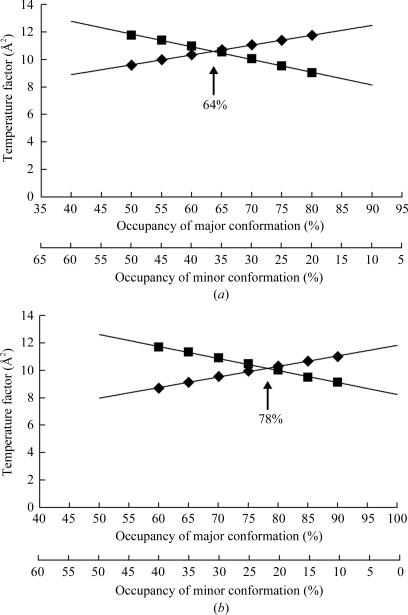
Plots of occupancy *versus* temperature factor of the two Thr101 side-chain conformers in the unsoaked (*a*) and soaked (*b*) structures. The plotted temperature factors were the average of three atoms (C^β^, C^γ^ and O^γ^ atoms) of the Thr101 side chain. Diamonds, rotamer represented by the major-component structure; squares, rotamer represented by the minor-component structure. In the unsoaked structure, occupancies of 64 and 36% for the major and minor rotamers, respectively, gave the same temperature factors; in the soaked structure, occupancies of 78 and 22% for the major and minor rotamers, respectively, provided the same temperature factors.

To determine the occupancy of each Thr101 side-chain rotamer, we refined the structures of the soaked and unsoaked crystals under several different occupancy values. Refined temperature factors for the Thr101 side chain (the average of those for C^β^, C^γ^ and O^γ^ atoms) were found to correlate highly with occupancy (Figs. 3 ▶ *a* and 3 ▶ *b*) and we assumed that each rotamer would have the same temperature factor. When the occupancies of the rotamers represented by the major- and minor-component structures of the unsoaked crystals were 66 and 34%, respectively, they have the same temperature factor of 10.6 Å^2^ (Fig. 3 ▶ *a*). Similarly, the occupancies of the major and minor rotamers of the soaked crystal were determined as 78 and 22%, respectively (Fig. 3 ▶ *b*).

**Figure 4:**
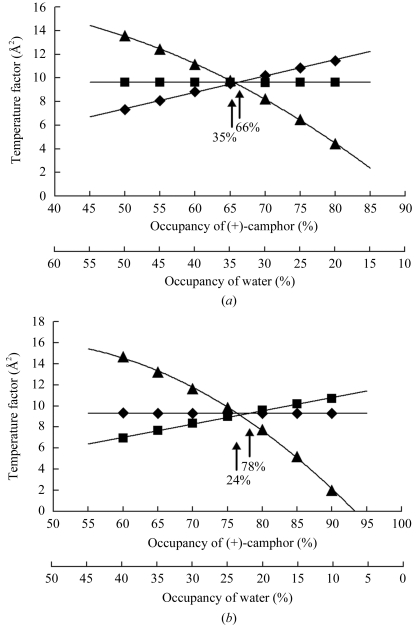
Plots of occupancy *versus* temperature factor of the active-site (+)-camphor and the water bound to the haem iron obtained by refinement of the unsoaked (*a*) and soaked (*b*) structures under the given value of occupancy (the occupancy of haem was fixed at 100%). Squares, (+)-camphor; diamonds, haem; triangles, water. The temperature factors for (+)-camphor and haem were the average of the whole molecules.

Refinements for the (+)-camphor molecule and the haem-bound water under several sets of occupancies were performed for the unsoaked and soaked crystals to determine their occupancies, assuming that the temperature factors of the (+)-camphor and water are almost equal to the temperature factor of the haem group. The occupancies and temperature factors are highly correlated (Figs. 4 ▶ *a* and 4 ▶ *b*). For the unsoaked crystal, when the respective occupancies of the (+)-camphor and the water were 66 and 35%, their temperature factors were almost equal to the averaged temperature factor of the haem group of 9.5 Å^2^ (Fig. 4 ▶ *a*). Since the (+)-camphor molecule cannot coexist with the water molecule, 35% occupancy of the water molecule is equivalent to 65% occupancy of the (+)-camphor molecule. Thus, the estimated occupancy of (+)-camphor in the unsoaked crystal is 65–66%. For the soaked crystal, occupancies of 78 and 24% for the (+)-camphor and water, respectively, resulted in temperature factors nearly equal to that of the haem group (Fig. 4 ▶ *b*). Consequently, the estimated occupancy of (+)-camphor in the soaked crystal is 76–78%.

These analyses indicate that the occupancy of the major Thr101 rotamer is almost identical to that of the occupancy of (+)-camphor and that that of the minor Thr101 rotamer is almost equal to that of the water molecule. These agreements imply that (+)-camphor binding to the active site changes the conformation of the Thr101 side chain from that of the minor component to that of the major component. Thus, the structure of the major component shown in Fig. 2 ▶(*b*) is the structure of (+)-camphor-bound P450cam, while the structure of the minor component shown in Fig. 2 ▶(*a*) is the (+)-camphor-free (water-bound) structure. Our substrate-free structure superposes well with the previously reported structure of Poulos *et al.* (1986 ▶). In the free structure, it has been reported that six water molecules, including a water molecule bound to haem, are located in the substrate-binding site. Such water molecules are also suggested in this study by observed electron density other than that of the bound (+)-camphor both in the soaked and unsoaked crystals.

The present study indicates that Thr101 hydrogen bonds to Tyr96 in the camphor-free state (Fig. 2 ▶ *a*) and changes conformation upon (+)-­camphor binding to form a hydrogen bond to the peripheral 6-­propionate of haem (Fig. 2 ▶ *b*). Since Thr101 functions as the hydrogen donor in the hydrogen bond, it raises the redox potential of the haem iron. This conformational change of Thr101 together with the spin-state change contributes to the efficiency of (+)-camphor hydroxylation catalyzed by this enzyme.

## Supplementary Material

PDB reference: cytochrome P450cam, unsoaked, 2zwt, r2zwtsf

PDB reference: (+)-camphor-soaked, 2zwu, r2zwusf

## Figures and Tables

**Table 1:** Data-collection, processing and refinement statistics Values in parentheses are for the highest resolution shell.

	Unsoaked	Soaked
Data collection		
X-ray source	SPring-8 BL41XU	SPring-8 BL44XU
Wavelength (Å)	0.8	0.7
Space group	*P*4_3_2_1_2	*P*4_3_2_1_2
Unit-cell parameters (Å)	*a* = *b* = 63.38, *c* = 247.30	*a* = *b* = 63.61, *c* = 250.39
Resolution (Å)	50.00–1.35 (1.40–1.35)	50.00–1.30 (1.35–1.30)
Observed resolutions	779984 (80388)	610735 (61650)
Unique reflections	113101 (11165)	125182 (12,324)
Completeness (%)	99.5 (100.0)	98.6 (98.9)
Redundancy	6.9 (7.2)	4.9 (5.0)
Average *I*/σ(*I*)	41.4 (4.8)	34.4 (4.9)
*R*_merge_†	0.064 (0.400)	0.075 (0.407)
Refinement		
*R* factor‡ (%)	16.3	16.6
*R*_free_‡ (%)	19.0	18.4
R.m.s. deviation from ideal values		
Bond lengths (Å)	0.010	0.010
Bond angles (°)	1.5	1.5

† *R*_merge_ = 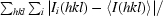 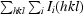, where 〈*I*(*hkl*)〉 is the mean intensity of *i* reflections with intensities *I*_*i*_(*hkl*) and common indices *hkl*.

‡ *R* factor = 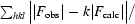 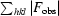, where *F*_obs_ and *F*_calc_ are the observed and calculated structure factors, respectively. *R*_free_ was calculated for a randomly chosen 5% of reflections and *R* factor was calculated for the remaining 95% of reflections.
